# The Knowledge, Attitude and Practice Among Makkah Physicians Towards Herpes Zoster Vaccination, Saudi Arabia, 2023

**DOI:** 10.7759/cureus.49393

**Published:** 2023-11-25

**Authors:** Salman A Turkistani, Faten J Althobaiti, Sami H Alzahrani

**Affiliations:** 1 Preventive Medicine, Ministry of Health, Makkah, SAU; 2 Preventive Medicine, Saudi Board of Preventive Medicine, Makkah, SAU

**Keywords:** vaccination, herpes-zoster, makkah, physicians, practice, attitude, knowledge

## Abstract

Background

Herpes zoster (HZ) is a viral disease, which is more common among the elderly and immunodeficient individuals, among which approximately 22% of cases might progress to post-herpetic neuralgia (PHN). Hence, this study aimed to assess the knowledge, attitude and practice (KAP) towards HZ and its vaccination among primary health care physicians in Makkah, Saudi Arabia, 2023.

Methodology

This analytical cross-sectional study used an online pre-validated questionnaire and was conducted from July to August 2023. The target population included physicians working in primary healthcare (PHC) daily clinics in Makkah.

Results

A total of 153 participants were included in the current study. Of which 90 (58.8%) were females and 120 (78.4%) participants had chicken pox history. Around 123 (80.4%) had previously heard about shingles. The most reported source of information was physicians (63%) followed by the Internet (12.2%). Risk factors for shingles were found to be immunodeficiency (95.1%) and age (78%). Most (88.2%) participants had previously heard about the shingles vaccine and 99 (64.7%) reported that the shingles vaccine is needed even if the patient had chicken pox in the past. Most participants (82.4%) knew that the vaccine should be given to adults aged more than 50 years. About 69 (45.1%) thought that they were extremely likely to get the shingles vaccine if the doctor recommended it. Barriers to shingles vaccination among study participants included participant's perception that they were not at risk of getting shingles (33%) and concerns about vaccines’ side effects (27.5%). The average knowledge score about shingles was found to be 9.51 ± 3.14 and the average knowledge score about shingles vaccine was found to be 5.43 ± 1.46. Gender was significantly associated with knowledge score about the vaccine (p-value= 0.028) where females had higher knowledge scores about shingles vaccine as compared to males. Qualification level and current Saudi Commission for Health Specialties (SCFHS) classification were found to be significantly associated with knowledge scores about shingles (p-value = 0.002 and 0.003, respectively).

Conclusion

A good level of KAP about shingles and its vaccine was found among the study participants. However, few knowledge gaps in methods of protection were assessed. Female gender, married participants and higher SCFHS qualification level were positively associated with higher levels of knowledge and awareness as compared to other groups.

## Introduction

Varicella-Zoster Virus Reactivation causes Herpes Zosters (HZ) an infectious disease, also known as shingles. The virus stays dormant in the dorsal root ganglia and is characterized by the cluster appearance of herpes along the peripheral nerves on one side of the body [1]. HZ is more common among the elderly and people with immunodeficiency, among which approximately 22% of cases might progress to post-herpetic neuralgia (PHN). The pain can last for months and even years and severely affects the quality of life [2]. HZ commonly presents as a vesicular dermatological rash that does not cross the body’s midline and crusts within 10 days. The pain associated with the rash can be highly variable; patients might experience hypersensitivity, tingling, aching, or burning pain [3].

The estimated HZ incidence among the Western countries’ population has been rising to 3-5 per 1000 individuals every year. With advancing age, the chances of developing HZ are also increasing, with up to 50% chances above 85 years of age [4]. Thus, 10-20% of patients with HZ aged ≥ 50 years develop PHN. The female population is more prone to develop HZ as compared to the male population. The incidence also increases with compromised immunity [5]. It has been observed that with increasing age, the chances of developing this disease have significantly increased along with PHN. The severity of HZ is an alarming indication of the increased burden on the national healthcare systems [6].

The infection due to HZ has been treated with antiviral drugs along with corticosteroids but treatment of PHN with drugs is still under research. PHN is highly resistant to treatment as management is limited to antiviral treatment and analgesics. Few medicine groups have shown some beneficial effects on improving pain and quality of life. Drugs like opioids, anticonvulsants and tricyclic antidepressants might be effective against neuropathic pain. Acupuncture, chilli-based creams and psychological support for depression and pain management have also been used with positive effects [7].

A prophylactic vaccine that was introduced in 2006 and the use of the above-mentioned drugs have shown a significant improvement in reducing the mortality rate and helped to reduce the severity of the disease in all affected age groups [8]. Administering two doses of HZ vaccine reduced the prevalence and severity of HZ and PHN. The vaccination also helped individuals with any immune-compromised condition or sensitivity. The vaccine administration to the older population showed a reduction in the incidence of HZ by 51%. It also showed a 90% role in reducing the chance of occurrence in immunocompromised populations. In the absence of an HZ vaccine, it has been estimated that around 20% to 30% population and about 50% of those living at the age of 85 years would develop HZ [9].

Multiple researches have been conducted in many countries and have proved the effectiveness of the vaccine. A cross-sectional study concluded that misconceptions about HZ were notable among the study population. More health education is needed to improve the understanding and enhance awareness of HZ among the public [10]. Another study revealed that attitudes towards the HZ vaccine were generally positive; however, due to a lack of knowledge, poor practices were observed [11]. One more study concluded that not only increased knowledge but also a change in attitude and practice is needed to enhance the implementation of national recommendations [12]. Another study showed that HZ vaccination rates remain relatively low when compared with rates of influenza and pneumonia vaccination [13]. Furthermore, a cross-sectional survey concluded that educational programs are needed for parents to improve knowledge about vaccination and immunization coverage [14,15]. Another study concluded that the general Saudi population had a good understanding of HZ and its vaccine, although their attitudes towards the HZ vaccine were generally positive but poor practices were observed [16]. Moreover, studies have also urged to start more awareness programs in university’ campuses to educate students on methods of prevention [17]. Another study showed the effectiveness of recombinant zoster vaccine (RZV) for preventing herpes zoster ophthalmicus (HZO), whereas regarding low vaccination rate, the public health need was highlighted to increase HZV use [18]. Another study stated that individual attitudes towards zoster vaccination are closely related to subjective perceptions of HZ and views on vaccination in general [19].

This cross-sectional study aimed to assess the knowledge, attitude and practice (KAP) towards HZ and its vaccination among primary health care physicians in Makkah, Saudi Arabia, 2023.

## Materials and methods

Study design

This was an analytical cross-sectional study conducted during the period from July to August 2023.

Inclusion and exclusion criteria

Registered physicians at the Saudi Commission for Health Specialties (SCFHP) including the general practitioners (GPs) and family physicians (consultants, specialists and residents) working in primary healthcare (PHC) centres of the Ministry of Health in Makkah City, Saudi Arabia during the proposed study period were included after obtaining the informed consent from them. However, physicians volunteering for work, interns, and physicians who did not speak English and who did not consent to take part in the study were excluded.

Data collection tool

The data collection tool was obtained from another study and was assessed and checked to ensure content reliability and face validity by a biostatistician [11]. The data collection was done through a self-administrated questionnaire composed of a total of 33 questions, which was created on Google Forms (a web-based copy form) (Google, Inc., Mountain View, CA, USA). The form was distributed to the healthcare physicians in the PHC centres in Makkah on their mobile phones through an online link by SMS and the response was collected automatically into an Excel Sheet (Microsoft® Corp., Redmond, WA, USA). When required, communication between the investigator and the study participants was established via electronic mail.

The form was in English language and was pre-tested for clarity and ease of use. Each question in the form was mandatory to be answered to avoid missing values at the data analysis stage.

A pilot study was conducted on 5-10% of the calculated sample size to test for the clarity of the questions, the appropriateness of the online form and the time required to finish the questionnaire before starting the actual research. The participants of the pilot study were not included in the final data analysis.

The data collection was done using a self-administered questionnaire. The questionnaire was divided into three sections. The first section (1-9) included questions on demographic data (gender, nationality, age, marital status, qualification and classification level, disease history, and history of chickenpox infection). The second section (10-18) included six questions that were related to knowledge about chicken pox, shingles and their risk factors. Questions 13, 14 and 15 were related to the fear of getting chickenpox and shingles, the fear of getting the disease from others and the fear of no cure. From 16 to 26 all questions were related to shingles, its signs and symptoms, and effects. The third section (19-33) included questions on the HZ vaccine, knowledge and perception about the vaccine, suggestions about the administration of the vaccine in specific age groups and how to prevent exposure from an affected person. In the last section, questions from 27 to 32 were about knowledge of the disease, fear of getting it, effects of the disease on the body, how to prevent it, cost of the disease and doctor comments and recommendations about this disease. The last 33rd question contained the perception of not getting the disease and a possible way out.

Sampling technique and sample size calculation

A multistage stratified sampling technique was used. Stratification of PHC centres into five sectors as regulated by the executive administration of primary care. Then the sample was selected randomly from each sector using a simple random technique. The total number of healthcare workers (HCWs) in Makkah City was 4,869, according to the last Ministry of Health yearly statistical book 1440 [20]. The estimated number of HCWs was around 1800 roughly, while the estimated number of physicians was 250. The sample size was calculated to be 125 at a 95% confidence interval and 5% margin of error.

Variables

The dependent and independent variables of the study include the knowledge variables and sociodemographic variables, respectively (Table 1).

**Table 1:** Dependent and independent variables

Dependent variables	Independent variables
Total knowledge about shingles (10-19)	Sociodemographic variables (age, gender, nationality)
Total knowledge about shingles vaccine (20-25)	Level of qualification and classification
Total scores of attitudes and practice (26-33)	

Statistical analysis

The statistical analysis was done using the Statistical Package for the Social Sciences (IBM SPSS Statistics for Windows, IBM Corp., Version 21, Armonk, NY). Mean and standard deviation were used to summarize continuous variables while proportions, percentage and frequency tables were used to summarize the categorical variables. Student t-tests and analysis of variance (ANOVA) were used to find significant differences between the groups. Chi-square and Fisher's exact test were used to find significant associations between categorical variables. The significance level was less than 0.05 and 95% for a confidence interval.

Ethical considerations

All participants’ information was confidential and used only in the study research process. The investigator will describe the aim and objectives of the study to the HCWs, and ask them to provide consent after they receive all needed information. A verbal informed consent was obtained from all the participants. The study protocol was approved by the Research Committee of the Saudi Program of Preventive Medicine in Makkah and the Ethical Research Committee of the Health Directorate in Makkah (IRB Number: H-02-K-076-0623-992).

## Results

About 153 participants were included in the current study. Around 63 (41.2%) were males. All the participants were less than 50 years old. Eighty-four (54.9%) participants were married. Almost 150 (98%) were Saudi. The place of residence was found to be Makkah for about 141 (92.2%) participants. The last qualification level was found to be family medicine board for 81 (52.9%) participants. Around 36 (23.5%) participants were found to be current SCFHC residents. The most commonly reported chronic disease among study participants was asthma (11.8%), followed by hypercholesterolemia (5.9%) and hypothyroidism in nine (5.9%) (Table 2).

**Table 2:** Socio-demographic characteristics of the study participants (n=153) SCFHS: Saudi Commission for Health Specialties

Variable	Categories	Frequency	Percent
Gender	Male	63	41.2
	Female	90	58.8
Age (in years)	< 50	153	100
Nationality	Saudi	150	98
	Non-Saudi	3	2
Marital status	Single	60	39.2
	Married	84	54.9
	Divorced	9	5.9
Place of residence	Makkah	141	92.2
	Jeddah	12	7.8
What is your last qualification level?	Bachelor of Medicine and Surgery (MBBS)	63	41.2
	High Diploma	3	2
	Master degree	3	2
	PhD	3	2
	Family Medicine Board Certification	81	52.9
Current SCFHS classification	Resident	36	23.5
	Training resident	36	23.5
	Registrar	18	11.8
	Senior registrar	39	25.5
	Consultant	24	15.7
History of chronic diseases	None	99	64.7
	Hypertension	6	3.9
	Diabetes	6	3.9
	Hypercholesterolemia	9	5.9
	Hypothyroidism	9	5.9
	Gout	3	2
	Osteoarthritis	3	2
	Depression	6	3.9
	Asthma	18	11.8
	Others	6	3.9

About 120 (78.4%) participants had chicken pox history. Almost 123 (80.4%) have previously heard about shingles. The source of information was the physician (63%) followed by the Internet (12.2%). Risk factors for shingles were found to be immunodeficiency (95.1%), followed by age (78%). About 93 (60.8%) participants knew that getting chicken pox is a risk factor for shingles. Around 90 (58.8%) participants mentioned that they could get shingles if they were exposed to somebody with shingles. About 111 (72.5%) participants disagreed with the statement, which stated that there is no cure for shingles. According to the findings reported by the participants, shingles affected these groups more frequently than others; the elderly were the most frequently affected as stated by 120 (78.4%) participants, immunocompromised as stated by 114 (74.5%) participants, female gender as reported by 48 (31.4%) and pregnancy as mentioned by 39 (25.5%) participants. The most commonly reported symptoms, signs and complications of shingles were rash as stated by 147 (96.1%) participants, neuropathic pain mentioned by 123 (80.4%) participants, and blisters as reported by 120 (78.4%) participants. About 135 (88.2%) participants have previously heard about the shingles vaccine. The source of information about the shingles vaccine was the doctor as mentioned by 108 (70.6%) participants followed by the Internet as reported by 27 (17.6%) participants (Table 3).

**Table 3:** Knowledge about shingles risk factors, signs, symptoms and complications

Variable	Categories	Frequency	Percent
Have you ever had chicken pox?	Yes	120	78.4
	No	33	21.6
Have you ever heard of shingles?	Yes	123	80.4
	No	30	19.6
If yes, how did you hear about shingles? (n=123)	Doctor	78	63.4
	Heard about it from someone	3	2.4
	Know someone who had shingles	15	12.2
	Internet	15	12.2
	I had shingles	3	2.4
	Other	9	7.3
What do you think are risk factors for shingles? (n=123)	Unhealthy diet	15	12.2
	Chronic diseases	72	58.5
	Age	96	78
	Not getting enough sleep	15	12.2
	Gender	18	14.6
	Stress	63	51.2
	Immunodeficiency	117	95.1
For the following statements, choose the most relevant option:			
If I get chicken pox, I am at more risk of getting shingles	True	93	60.8
	False	21	13.7
	I don't know	39	25.5
I could get shingles if I come into contact with somebody who has it	True	90	58.8
	False	45	29.4
	I don't know	18	11.8
There is no cure for shingles	True	24	15.7
	False	111	72.5
	I don't know	18	11.8
What groups of people do shingles affect?	Children	21	13.7
	Elderly	120	78.4
	Males	33	21.6
	Females	48	31.4
	Immunocompromised	114	74.5
	Pregnant women	39	25.5
	All age groups	42	27.5
What are the signs, symptoms and complications of shingles?	Rash	147	96.1
	Blisters	120	78.4
	Cough	9	5.9
	Fever	90	58.8
	Sore throat	15	9.8
	Neuropathic pain	123	80.4
	Blindness	60	39.2
Have you ever heard of the shingles vaccine?	Yes	135	88.2
	No	18	11.8
If yes, how did you hear about the vaccine?	Doctor	108	70.6
	Vaccination schedule	12	7.8
	Internet	27	17.6
	Other	3	2
	I don't know	3	2

About 132 (86.3%) participants agreed with the statement, which stated that shingles vaccine could reduce the incidence of the disease by more than 50%. The majority (82.4%) disagreed with the statement that shingles vaccine could treat active disease. About 99 (64.7%) participants reported that a shingles vaccine is needed even if the patient had chicken pox in the past. Most participants (82.4%) knew that the vaccine should be given to adults aged more than 50 years. When encountering someone infected with shingles, 90 (58.8%) participants mentioned being vaccinated as a form of protection (Table 4).

**Table 4:** Knowledge about shingles vaccine

Variable	Categories	Frequency	Percent
For the following statements, choose the most relevant option:			
Shingles vaccine can reduce the incidence of disease by more than 50%	True	132	86.3
	False	6	3.9
	I don't know	15	9.8
Shingles vaccine can treat active shingles	True	3	2
	False	126	82.4
	I don't know	24	15.7
Shingles vaccine is not needed if the person already had chicken pox as a child	True	9	5.9
	False	132	86.3
	I don't know	12	7.8
Shingles vaccine is no longer needed if the person already had shingles	True	18	11.8
	False	99	64.7
	I don't know	36	23.5
The shingles vaccine should be given to:	Infants (under 1)	3	2
	Children (1-18)	3	2
	Adults (18-50)	0	0
	Adults (50+)	126	82.4
	All age groups	21	13.7
When you encounter someone infected with shingles, how do you protect yourself?	Wear a mask	39	25.5
	Do not share food	51	33.3
	Do not hug or shake hands	87	56.9
	Do not use the same utensils	81	52.9
	Take medications	18	11.8
	Get vaccinated	90	58.8
	Do nothing	30	19.6

In regards to worrying about getting shingles, 12 (7.8%) participants mentioned it as extremely likely. Around 75 (49%) participants thought it likely that shingles has a significant effect on health. Almost 54 (35.3%) participants were extremely likely that they were interested in knowing more about the disease. About 69 (45.1%) thought that they were extremely likely to get the shingles vaccine if the doctor recommended it (Table 5).

**Table 5:** Participants' attitudes and practice toward shingles

For the following statements, choose the most relevant option:	Extremely likely	Likely	Neutral	Unlikely	Extremely unlikely
I am worried about getting shingles	12 (7.8%)	24 (15.7%)	78 (51%)	18 (11.8%)	21 (13.7%)
Shingles has a significant effect on health	45 (29.4%)	75 (49%)	30 (19.6%)	3 (2%)	0 (0%)
I am interested in knowing more about this disease	54 (35.3%)	63 (41.2%)	30 (19.6%)	6 (3.9%)	0 (0%)
I am interested in knowing about how to prevent it	66 (43.1%)	54 (35.3%)	27 (17.6%)	6 (3.9%)	0 (0%)
I worry about the cost of the vaccine	15 (9.8%)	21 (13.7%)	48 (31.4%)	48 (31.4%)	21 (13.7%)
I would get the shingles vaccine if the doctor recommended it	69 (45.1%)	60 (39.2%)	21 (13.7%)	0 (0%)	3 (2%)

**Figure 1:**
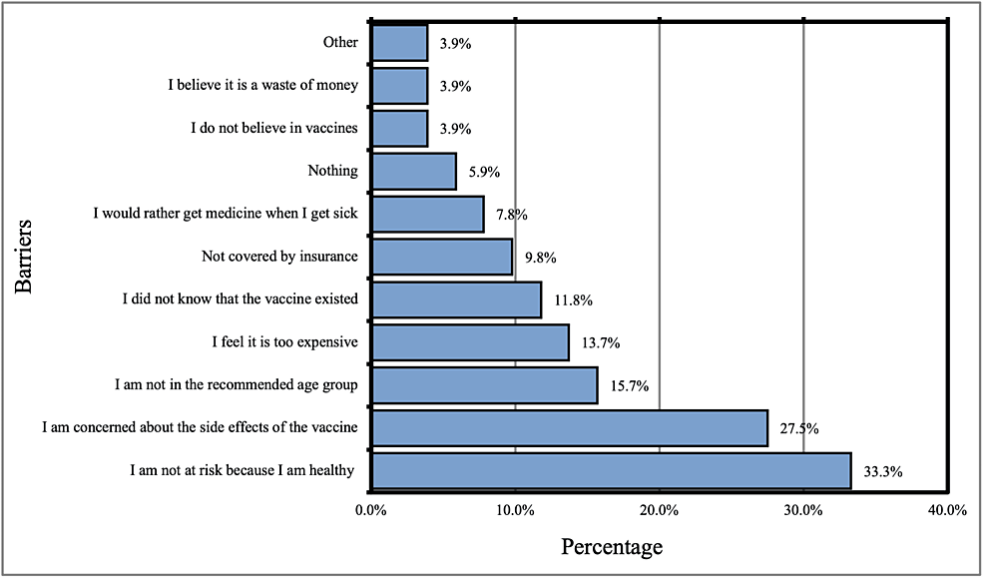
Barriers that prevent participants from getting the shingles vaccine

Barriers to shingles vaccination among study participants include participant's perception that they were not at risk of getting shingles (33%). In about 27.5% of participants, the barrier was concerned about the vaccine’s side effects (Figure 1).

Out of 16, the average knowledge score about shingles was found to be 9.51 ± 3.14 and the average knowledge score about shingles vaccine was found to be 5.43 ± 1.46 out of a total of eight. Further, gender was not found to be significantly associated with awareness about shingles (p-value = 0.165), but gender was significantly associated with knowledge score about vaccine (p-value = 0.028), with female gender tend to have higher awareness about shingles vaccine compared to males. A significant association was found between marital status and awareness about shingles (p-value = 0.001) and awareness about shingles vaccine, where divorced and married groups were found to have higher awareness levels when compared to singles. Qualification level and current SCFHS classification were found to be significantly associated with knowledge scores about shingles (p-value = 0.002 and 0.003, respectively). A statistically significant association was found between history of chronic diseases and awareness about shingles (p-value< 0.001) with those who had no history of chronic diseases tended to have higher awareness compared to participants with history of chronic diseases (Table 6).

**Table 6:** Factors associated with knowledge about shingles and the shingles vaccine SCFHS: Saudi Commission for Health Specialties

Variable	Awareness about shingles		Knowledge score (of the disease)		Awareness about the shingles vaccine		Knowledge score (of the vaccine)	
	Yes, n=51	P value	Mean (SD)	P value	Yes, n=51	P value	Mean (SD)	P value
Gender								
Male	54 (85.7%)	0.165	9.71 (3.55)	0.502	54 (85.7%)	0.418	5.10 (1.81)	0.028*
Female	69 (76.7%)		9.37 (2.83)		81 (90%)		5.67 (1.11)	
Nationality								
Saudi	120 (80%)	0.615	9.48 (3.16)	<0.001*	132 (88%)	1.000	5.40 (1.46)	< 0.001*
Non-Saudi	3 (100%)		11.00 (0)		3 (100%)		7.00 (0)	
Marital status								
Single	39 (65%)	< 0.001*	8.80 (3.37)	0.018*	48 (80%)	0.033*	5.05 (1.37)	0.012*
Married	75 (89.3%)		9.79 (2.93)		78 (92.9%)		5.61 (1.51)	
Divorced	9 (100%)		11.67 (2.18)		9 (100%)		6.33 (1.00)	
Place of residence								
Makkah	114 (80.9%)	0.704	9.55 (3.23)	0.559	123 (87.2%)	0.362	5.55 (1.45)	< 0.001*
Jeddah	9 (75%)		9.00 (1.65)		12 (100%)		4.00 (0.74)	
What is your last qualification level?								
Bachelor of Medicine & Surgery (MBBS)	45 (71.4%)	0.137	8.90 (3.36)	0.002*	57 (90.5%)	0.004*	4.90 (1.32)	< 0.001*
High Diploma	3 (100%)		12.00 (0)		3 (100%)		7.00 (0)	
Master degree	3 (100%)		11.00 (0)		3 (100%)		6.00 (0)	
PhD	3 (100%)		4.00 (0)		0 (0%)		1.00 (0)	
Family Medicine Board Certification	69 (85.2%)		10.04 (2.81)		72 (88.9%)		5.93 (1.19)	
Current SCFHS classification								
Resident	24 (66.7%)	0.004*	8.08 (3.45)	0.003*	30 (83.3%)	0.158	4.67 (1.57)	0.005*
Training resident	33 (91.7%)		10.83 (1.80)		36 (100%)		5.50 (0.97)	
Registrar	18 (100%)		10.00 (3.20)		15 (83.3%)		5.50 (2.64)	
Senior registrar	27 (69.2%)		9.08 (3.61)		33 (84.6%)		5.77 (1.06)	
Consultant	21 (87.5%)		10.00 (2.50)		21 (87.5%)		5.88 (0.80)	
History of chronic diseases								
Yes	33 (61.1%)	< 0.001*	8.56 (3.69)	0.011*	45 (83.3%)	0.165	5.28 (1.57)	0.339
No	90 (90.9%)		10.03 (2.67)		90 (90.9%)		5.52 (1.40)	

## Discussion

Assessing KAP regarding HZ vaccination is significant as the role of the doctor in knowledge distribution and patient's awareness depends on the level of awareness of the doctor. In addition, knowledge about shingles vaccine is important in certain situations in which any particular patient should be advised to get a shingles vaccination shot or not [20].

More than half (58.8%) of participants were females and 54.9% of participants were married. The vast majority (98%) of the participants were Saudi nationals. Family medicine was the last qualification level for more than half (52.9%) of the participants.

More than two-thirds (78.4%) of participants had chicken pox before but the infection rate was reported in the other study to have reached 90% [21] and this could be attributed to differences in the studied samples. The vast majority (80.4%) had previously heard about shingles and this percentage was higher when compared to the findings reported in the parallel study conducted by Al-Khaldi, et al., in which 64.3% of participants had heard about shingles and this variability in the initial knowledge could be due to different demographic variables and environmental factors [11]. More than half (60.8%) of participants were aware that getting chicken pox is a risk factor for shingles. The most frequently reported risk factor for shingles was immunodeficiency as stated by the greatest majority (95.1%) of the participants. This result was likely to occur as it is linked with the general knowledge about the disease which is more prevalent in the elderly and those with decreased immunity.

Further, age was reported by more than two-thirds (78%) of the participants, and chronic diseases by more than half (58.5%) of the participants and this was found to be consistent with the findings reported in the previous study [22]. The majority (88.2%) of the participants had previously heard about the shingles vaccine and this percentage was much higher than the percentage found in the congruent study carried out by Yang Tu as only 43.6% of participants had heard about the shingles vaccine and this could be attributed to differences in the studied samples [23].

Most of the participants (86.3%) agreed with the statement that the shingles vaccine could reduce the incidence of the disease by more than 50%. Nearly two-thirds (64.7%) of the participants reported that the shingles vaccine is needed even if the patient had chicken pox in the past and this was found to be consistent with the findings reported in the study [16]. Most (82.4%) participants knew that the vaccine should be given to adults aged more than 50 years, similar findings were mentioned in the other study, which also reported that the vaccine should be given to those who are more than 50 years old [24].

About 13.8% were unlikely to get shingles vaccine, 13.7% were extremely unlikely to get shingles vaccine and about 45.1% thought that they were extremely likely to get shingles vaccine if the doctors recommend it. Further, 39.2% of participants would likely get shingles vaccine if their doctor recommended it. Analogous findings were reported in the study conducted by Baalabaki et al., in which 37.5% of participants were found willing to get vaccinated after a doctor’s recommendation [25].

Barriers to shingles vaccination among study participants included participant's perception that they were not at risk of getting shingles (33%). In about 27.5% of participants, the barrier was concerned about the vaccine’s side effects, this was different from the findings mentioned in one parallel study in which the main barriers were found to be the cost and availability [20].

Out of 16, the average knowledge score about shingles was found to be 9.51 ± 3.14 and the average knowledge score about shingles vaccine was found to be 5.43 ± 1.46 out of a total of eight. Regarding the association between shingles and participant's characteristics, qualification level and current SCFHS classification were found to be significantly associated with knowledge score about shingles. Gender was not found to be significantly associated with awareness about shingles, but was found significantly associated with knowledge scores about vaccines, with female gender tends to have higher knowledge scores about shingles vaccines compared to males. This was found similar to the findings of the other study in which the female gender was linked to increased knowledge and awareness about shingles and shingles vaccine [26].

The present study had some limitations to be addressed. First, the cross-sectional design might not reflect the true effect between the variables due to lack of temporality. While a good sampling technique was used, the study can be generalized only to the population and PHC physicians of Makkah City. The response rate was dependent upon the willingness of participants to fill out the form; a low response rate might affect the results of the study. Nevertheless, the benefits of this study included the assessment of KAP towards control and preventive measures of HZ among PHC physicians by using validated tools and knowing the necessity of interventions to improve the KAP of the physicians and the patient’s well-being. In addition, the study would be beneficial in identifying the gaps that might need further explanation in the clinical guidelines of HZ prevention, management and follow-up.

## Conclusions

A good level of KAP about shingles and shingles vaccines among the study participants was found. However, few knowledge gaps in methods of protection were assessed. Only a small proportion of the participants had previously been vaccinated against shingles. Barriers to vaccination were found to be the vaccine’s side effects and individual perceptions that they were not at risk of getting shingles. Female gender, married participants and higher SCFHS qualification level were found to be linked to higher levels of knowledge and awareness compared to other groups. Hence, the level of knowledge and awareness about shingles and shingles vaccines should be raised. Physicians should emphasize the importance of vaccination, especially in elderly patients and those who are immune compromised.
